# Spectral Cytometry Has Unique Properties Allowing Multicolor Analysis of Cell Suspensions Isolated from Solid Tissues

**DOI:** 10.1371/journal.pone.0159961

**Published:** 2016-08-08

**Authors:** Sandrine Schmutz, Mariana Valente, Ana Cumano, Sophie Novault

**Affiliations:** 1 Institut Pasteur, Flow Cytometry Core Facility, Paris, France; 2 Institut Pasteur, Immunology Department, Lymphopoiesis Unit, Paris, France; 3 Univ. Paris Diderot, Sorbonne Paris Cité, Cellule Pasteur, Paris, France; 4 Inserm U1223, Paris, France; 5 Instituto de Investigação e Inovação em Saúde (i3s) & INEB–Instituto de Engenharia Biomédica, Stem-Cell Microenvironments in Repair/Regeneration Team, Microenvironment for Newtherapies Group, Porto, Portugal; 6 ICBAS–Instituto de Ciências Biomédicas Abel Salazar, Universidade do Porto, Porto, Portugal; AntiCancer Inc., UNITED STATES

## Abstract

Flow cytometry, initially developed to analyze surface protein expression in hematopoietic cells, has increased in analytical complexity and is now widely used to identify cells from different tissues and organisms. As a consequence, data analysis became increasingly difficult due the need of large multi-parametric compensation matrices and to the eventual auto-fluorescence frequently found in cell suspensions obtained from solid organs. In contrast with conventional flow cytometry that detects the emission peak of fluorochromes, spectral flow cytometry distinguishes the shapes of emission spectra along a large range of continuous wave lengths. The data is analyzed with an algorithm that replaces compensation matrices and treats auto-fluorescence as an independent parameter. Thus, spectral flow cytometry should be capable to discriminate fluorochromes with similar emission peaks and provide multi-parametric analysis without compensation requirements. Here we show that spectral flow cytometry achieves a 21-parametric (19 fluorescent probes) characterization and deals with auto-fluorescent cells, providing high resolution of specifically fluorescence-labeled populations. Our results showed that spectral flow cytometry has advantages in the analysis of cell populations of tissues difficult to characterize in conventional flow cytometry, such as heart and intestine. Spectral flow cytometry thus combines the multi-parametric analytical capacity of the highest performing conventional flow cytometry without the requirement for compensation and enabling auto-fluorescence management.

## Introduction

Advances in flow cytometry (FCM) instrumentation and fluorochrome availability enabled a new era of polychromatic analysis. Among the most notable recent developments there is a substantial increase in fluorescent dyes available for cell phenotyping studies, in particular in fluorochromes excited by the violet laser (405nm), such as the Brilliant Violet and new Qdot dyes. However, the multiplication of fluorescent dyes increases the risk of overlapping emissions and requires labor-intensive compensation matrices in order to separate populations labeled with these multiple fluorochromes. Consequently, careful choice of fluorochrome combinations must be determined empirically and adapted to each experimental condition. Furthermore mouse models that use GFP and YFP as reporters of the expression of given proteins or lineage tracers of particular cell subsets, are difficult to analyze with conventional cytometers because these fluorescent dyes often emit in more than one detector. As FCM became widely used to analyze solid tissue cell suspensions auto-fluorescence, found either in the cells to be analyzed or in contaminating cells, limits the discrimination of specifically labeled populations.

The basic principles of the spectral FCM have been reported in Futamura et al [1]. The SP6800 spectral FCM equipped with 405/488/638nm lasers. The spectral FCM captures all the emitted sample fluorescence as spectra in 32-channel linear array PMT (32ch PMT) ranging from 500nm to 800nm and 2 independent PMTs ranging from 420nm to 440nm and from 450nm to 469nm, respectively, replacing the conventional band-pass filters. The 488 and the 405/638nm laser spots are spatially separated while the 405nm and 638nm laser spots are co-linear. For each single particle, the spectral FCM measures up to 66 channels of fluorescence data excited by 405nm and 488nm. When cells are excited by the 638nm laser the spectral FCM measures 58 channels of fluorescence data because a mask that shields light from 617nm to 662nm was inserted to prevent the 638nm laser from shining into the PMT.

The spectral FCM analyzes the acquired full spectrum data with algorithms based on the Least Squares Method (LSM) and on the Probabilistic Spectrum Analysis (PSA) that enable the separation of overlapping fluorescent spectra. Spectra derived from single stained and unstained samples are recognized as the basic reference spectra. Then, multi-stained samples are mathematically fitted and unmixed, a procedure whereby the measured spectrum of a sample, with mixed fluorescent labels, is decomposed into a collection of its constituent spectra, from each color being used and from the unstained reference spectra (single stained and unstained samples). Compensation that removes the signal from all detectors except the one measuring a given dye, in conventional FCM, is replaced by the deconvolution, in spectral technology [2,3].

Spectral FCM with its optical configuration and algorithm provides high sensitivity, automatic analysis and real-time spectral unmixing from all fluorescent signals in a multi-color analysis. Moreover, the spectral FCM captures and can remove auto-fluorescence of each cell, providing increased signal-to-noise ratio. These advances should allow performing high dimensional analyses of auto-fluorescent cells obtained from solid tissues.

In this study we used spectral technology to analyze the discriminative capacity in cell populations simultaneously labeled with dyes with very close emission spectra. We combined and tested 19 fluorochromes in a single 21-parameter analysis that characterized the major hematopoietic subsets found in the mouse spleen. Moreover, we show that spectral cytometry is particularly useful in the analysis of auto-fluorescent cells. In the characterization of intestinal cell suspensions where epithelial cells dominate, we could quantify complex and discrete populations of intra-epithelial lymphocytes and in embryonic heart, the auto-fluorescence management allowed assigning specific fluorescence to cells that would have otherwise been excluded from the analysis. Auto-fluorescence management was unique to spectral cytometry because in three other conventional cytometers used in parallel, our analysis was compromised irrespective of their optical configuration or constructer.

## Materials and Methods

### Experiment overview

C57BL/6 mice were purchased from Janvier Labs (France). To obtain timed-embryos we matted two females with one male overnight and the next morning vaginal plug was considered embryonic day 0.5 (E0.5). RoRγt-Cre:Rosa^FL-STOP-YFP^ [4,5] and Ubiquitin-GFP [6] were bred and obtained from the Pasteur Institute animal facility. Mice were euthanized by cervical dislocation. The Pasteur Institute Safety Committee in accordance with French Agriculture ministry and the EU guidelines approved all animal experiments.

### Flow sample and specimen description

Spleens were harvested, dissociated and resuspended in Hanks’balanced-salt solution (HBSS) supplemented with 1% FCS (Gibco). Small intestinal cells were isolated as previously described [7], except that the step of lymphocyte enrichment on a Percoll discontinuous gradient was omitted. Embryonic hearts were isolated and minced into small pieces (approximately 1 mm^3^) then sequentially digested with collagenase (C2139 Sigma, 100mg/ml) at 37°C for 15 minutes until no more tissue fragments were detected. Cell suspensions were then stained with pre-determined dilutions of antibodies for 20 minutes at 4°C, washed twice, filtered through a 70μM filter and resuspended in HBSS with 1% FCS with Propidium Iodide (PI, 0.5μg/ml). Cell samples not stained or stained with PI alone were always prepared. Cell sorting was done in a FACS Aria III (BD Biosciences). One Comp (eBiosciences) beads were individually stained with each antibody and washed twice before analysis. Antibodies were purchased from BD, eBiosciences, Biolegend, Sony or were a gift from P. Pereira (Pasteur Institute) (S1 Table).

### Quantitative RT-PCR

Cells were sorted in RLT Buffer (Qiagen) and were frozen at −80°C. RNA was obtained with an RNeasy Micro Kit (Qiagen), and cDNA was obtained with PrimeScript^TM^ RT Reagent Kit (Takara). A 7300 Real-Time PCR System (Applied Biosystem) and Taqman technology (Applied Biosystem) was used for quantitative RT-PCR. All values were normalized relative to GAPDH used as a house-keeping transcript. A bilateral unpaired Student's *t*-test was used for statistical analysis.

### Instrument details

Samples were analyzed in four different instruments. The Sony SP6800 (Sony Inc) has been previously described [1]. Instrument A is a FACSCanto II (BD Biosciences) equipped with three lasers (488, 650 and 405nm) and three pinholes with five PMT in the 488nm laser, two PMT in the 650nm laser and two PMT in the 405nm laser. Instrument B is a LSR Fortessa (BD Biosciences) equipped with four lasers (488, 575, 640 and 405nm) with four PMT in the 488nm laser, 4 PMT in the 575nm, 3 PMT in the 640nm and 6 PMT in the 450nm laser. Instrument C is a Cytoflex (Beckman Coulter) equipped with three lasers (488, 638 and 405nm), three sets of linear filters and 13 fluorescence detectors (PMT).

All instruments were calibrated with beads: CST beads for the BD instruments, daily and monthly calibration beads (Sony SP6800), Flowcheck beads (Cytoflex) before each experiment and data from the same cardiac and intestinal cell suspensions were sequentially acquired in the different instruments.

### Data analysis details

The analysis was performed using the Sony software (Sony, Inc), FlowJo 10.1r5 (Treestar, LLC USA) or the Kaluza1.2 or 1.5 softwares (Beckman Coulter) and statistical analysis (student’s t test) using Prisma Graph.

## Results

### Fluorescent probes with similar emission spectra can be efficiently separated in spectral FCM

**Fig 1:**
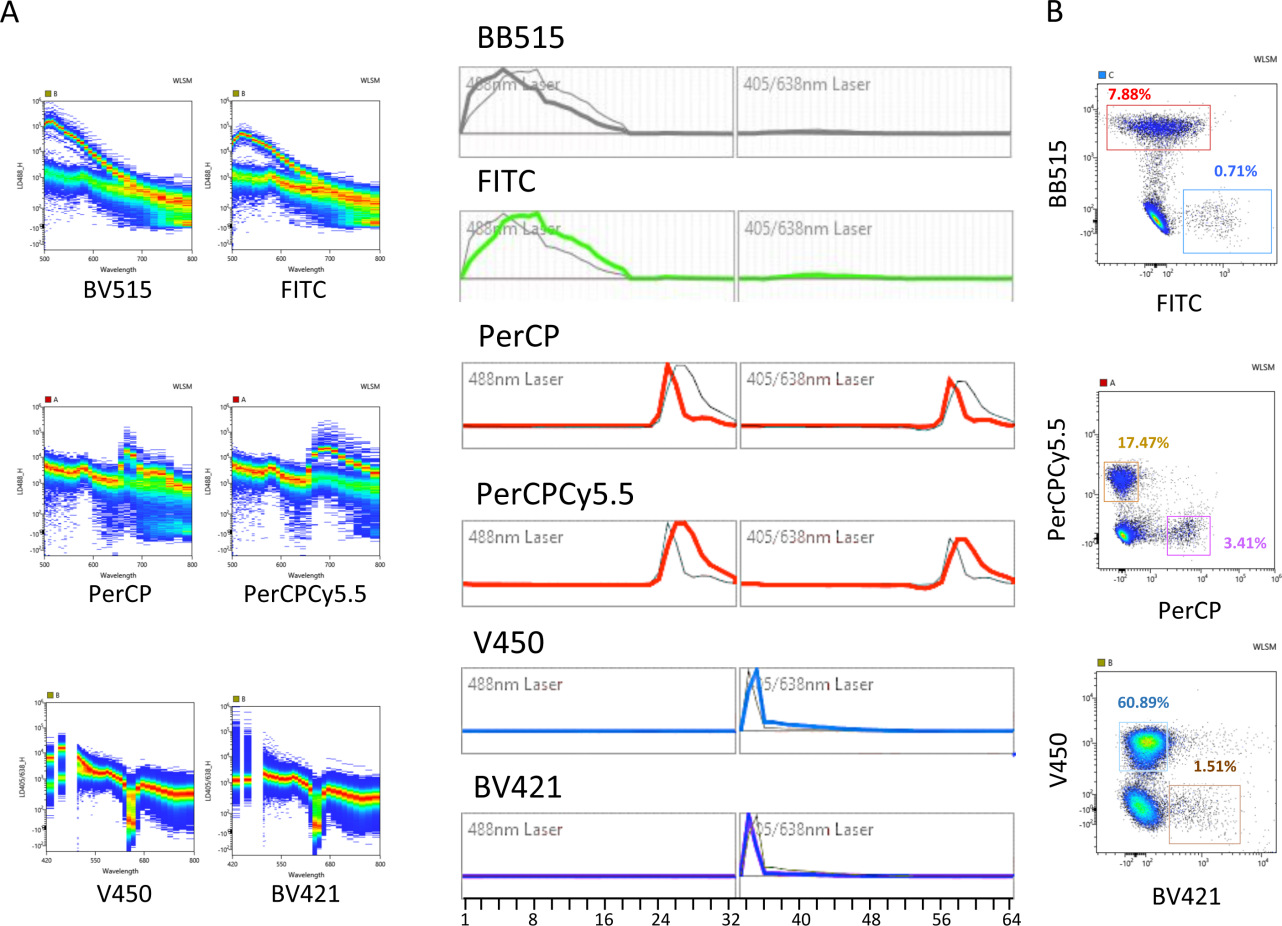
Spectral cytometry allows separating fluorescent probes with close emission peaks. Mouse splenic cells were labeled with fluorescence labeled antibodies. Two different representations of the spectra of dyes with very close emission wavelengths are shown after excitation with the blue: FITC-Ly49D (green) and BB515-CD8 (grey), PerCP-Gr-1 and Cy5.5-PerCP-IgM (red) or the violet laser: V450-IgD (blue) and BV421-CD11c (purple) (**A**) and can be discriminated on pseudo-color plots after the unmixing algorithm was applied (**B**).

**Fig 2:**
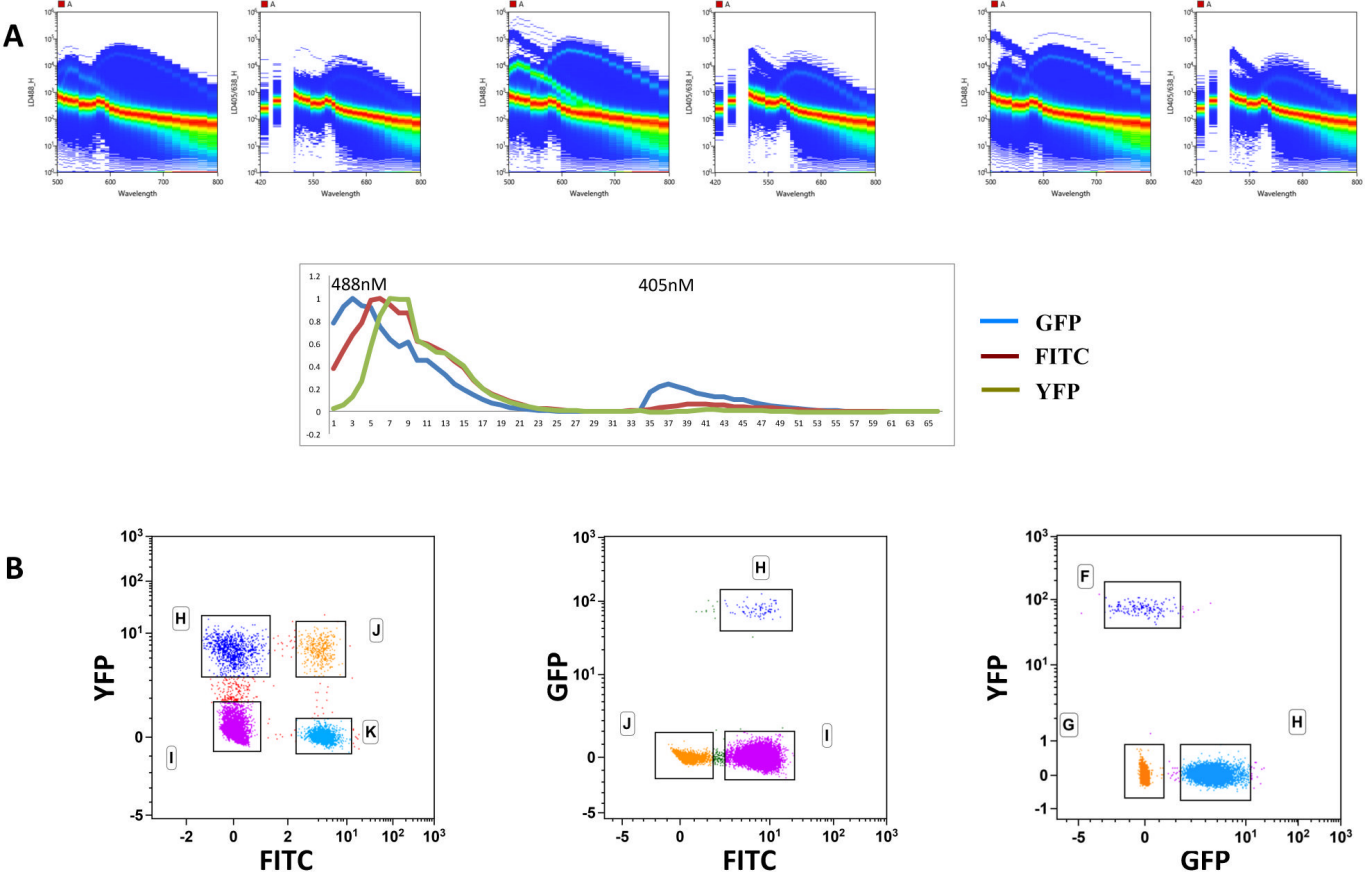
Spectral cytometry allows separating FTIC, from GFP and YFP. Splenic cells from wild type C57BL/6 mice, from a RoRγt-Cre:Rosa26^FL-STOP-YFP^ and from a ubiquitin-GFP CD8-TcR transgenic mouse were mixed two-by-two in similar proportions, stained with anti-CD8 FITC antibody and analyzed in spectral FCM. **A**. Plots showing the spectra on the two excitation lasers (left plot 488nm and right plot 405nm) of FITC with YFP and PI (left panels), FITC with GFP and PI (middle panels) and GFP with YFP and PI. The lower diagram shows the reference florochrome spectra shape calculated on single stained samples with GFP, FITC and YFP. **B**. Plots corresponding to the three independent unmixings of the two-by-two cell combinations. In all samples PI was added to exclude dead cells. The analysis was done in the Kaluza 1.5 software and the scales were readjusted in the FITC:YFP lower left plot.

In conventional FCM the analysis of populations labeled with fluorescent dyes with similar emission spectra requires special choices of mirrors and/or filters, often resulting in odd-shaped populations due to high compensation values and poor discrimination. We analyzed the capacity of spectral FCM to discriminate fluorochromes excited by the 488nm (Blue) or by the 405nm (Violet) lasers with largely overlapping emission wavelengths, impossible to combine in conventional FCM. We paired two-by-two in four independent cell samples antibodies labeled with the fluorescent dyes FITC (Ly49D) (Peak emission-PEm 518nm) with BB515 (CD8) (PEm 515nm), PerCP (Gr-1)(PEm 677nm) with Cy5.5-PerCP (IgM) (PEm 695nm), V450 (IgD) (PEm 457nm) and BV421 (CD11c) (PEm 423nm). Fig 1A shows the emission spectra of the fluorochromes analyzed individually in the two pinholes corresponding to the 488nm and to the 405nm lasers. BB515 and FITC have virtually identical PEm but exhibit somewhat different emission spectra, while BV421 and V450 have very similar spectra but differ in PEm by 36nm. PerCP and Cy5.5-PerCP have significant differences in both parameters. Fig 1B shows the dot plots obtained after the unmixing algorithm was applied to mouse splenic samples showing the ability of spectral FCM to discriminate dyes emitting in close wavelengths as BB515 and FITC (515 and 519nm). Moreover, we did not observe major distortions in the shape of the populations allowing unambiguous discrimination of distinct populations. Because several animal models report or trace cells expressing given proteins by the expression of GFP and/or YFP we tested the capacity of spectral FCM to distinguish cells marked with FITC (PEm 518nm), GFP (PEm 510nm) and YFP (PEm 529nm). We combined in the same sample splenic cells expressing two of these fluorescent probes in three independent combinations (Fig 2). GFP expression reported ubiquitin in CD8 TcR transgenic cells (0.5%) while YFP traced RoRγt, in innate lymphoid and αβT cells and FITC labeled CD8 T cells [4–6]. The top panels of Fig 2A show the fluorescence spectra of cells with PI labeled with YFP and FITC, in both 488 and 405nm laser (left panels), GFP and FITC (middle panels) and YFP and GFP (right panels) and the reference fluorochrome spectra shape calculated from single stained samples (lower panel). Fig 2B shows plots of the three (two-by-two) combinations of the labeled cells independently unmixed, showing that cells labeled with any of the three fluorochromes can be distinguished, albeit with different degrees of discrimination in any of the combinations. We concluded that spectral FCM was particularly suitable to distinguish fluorescent probes with similar PEm that cannot be associated in conventional FCM, although the association of several such fluorochromes can decrease the resolution of the fluorescence signal.

**Fig 3:**
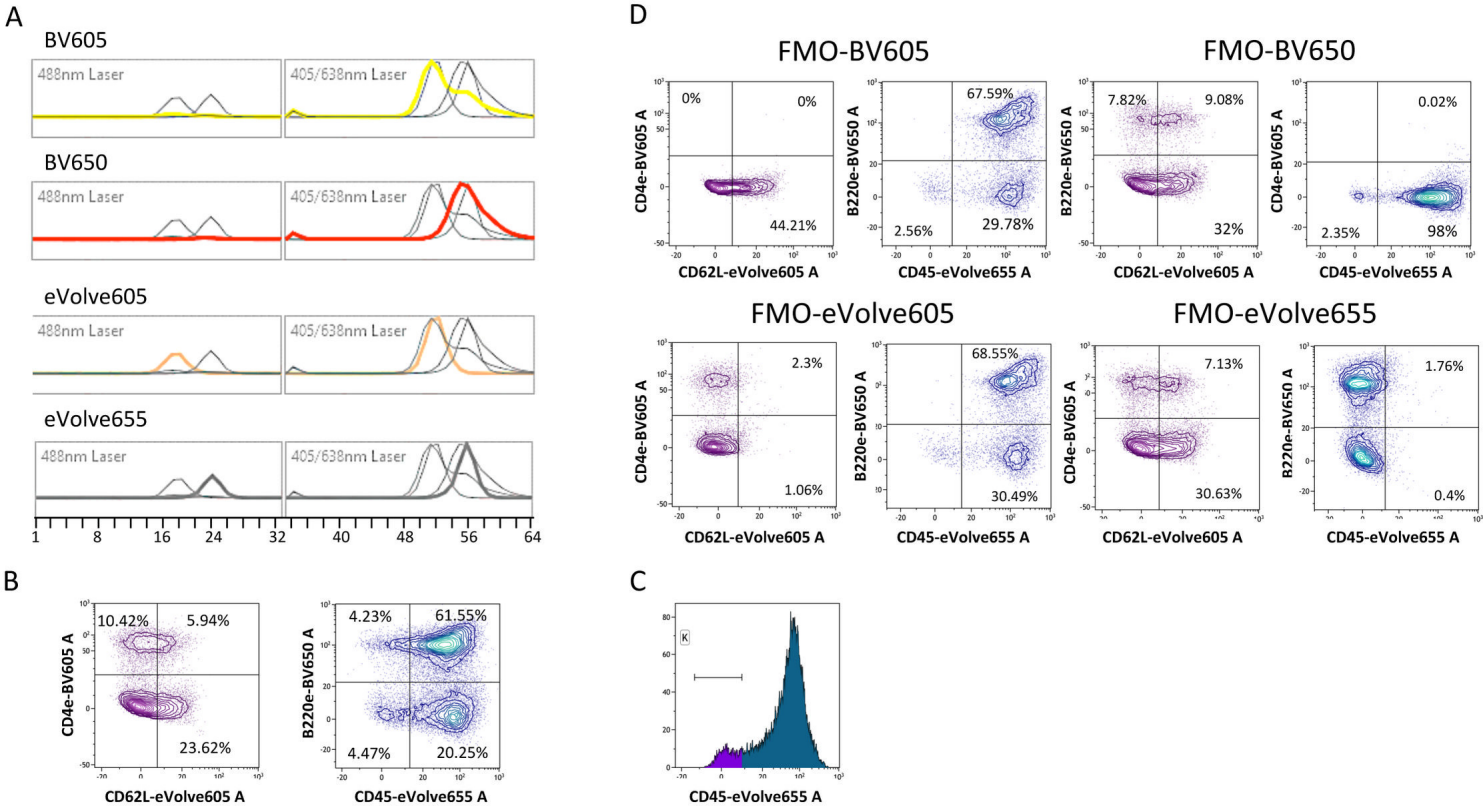
Spectral cytometry allows separating fluorescent probes with virtually identical peaks although with different shapes of the emission spectra. Small differences in the spectra shapes of BV605-CD4 (yellow) and eVolve605-CD62L (orange), and BV650-B220 (red) and eVolve655-CD45 (grey) (**A**) allow the discrimination between these four dyes when stainings were done with the four dyes together staining mouse spleen cells (**B**). **C.** Histogram showing the discrimination of CD45-eVolve655 positive from negative cells **D.** Fluorescence minus one (FMO) plots show the percentage of the populations when one dye is missing and the resulting strategy to place the quadrants. Data was analyzed in the Kaluza1.5 software.

We then analyzed whether dyes showing identical maximal photo-emission but with different shape spectra could be distinguished. We chose the combination of Sirigen polymer and Qdot dyes, BV605 (PEm 605nm), eVolve605 (PEm 605nm), BV650 (PEm 647nm) and eVolve655 (PEm 655nm). In Fig 3A the emission spectra of each fluorochrome in both laser spectra (488nm and the 405nm) is shown in color compared to the spectra of the other three fluorochromes in background grey. The eVolve^TM^ fluorochromes show narrower emission spectra than those of the Brilliant Violet^TM^ dyes and are cross-excited by the Blue laser. In order to verify the efficient discrimination of these four fluorochromes, we combined them in a sample of mouse splenic cells to analyze the expression of CD45 (eVolve 655), B220 (BV650), CD4 (BV605) and CD62L (eVolve605) (Fig 3B). Consistently, we found most splenic cells expressing CD45 (pan-hematopoietic marker) from which a large fraction is composed of B220^+^ cells (around 60%, marking B lymphocytes). CD4 T cells were normally represented (around 15%) with a fraction that varies according to age, sex and sanitary state of CD62L^+^ cells. The discrimination of CD45-eVolve655 positive and negative cells is shown in a histogram in Fig 3C. The fluorescence-minus-one (FMO) plots of the four fluorochromes are shown in Fig 3D, demonstrating specificity of the staining and showing the criteria to set the quadrants in the analysis. We concluded that, not only could spectral FCM discriminate fluorochromes with very similar emissions, but also fluorochromes with virtually identical peak emission wavelengths with small differences in the shape of the spectra.

### Twenty-one parameter spectral FCM panel

Multi-parametric FCM can be performed with different approaches using distinct technologies. Conventional analyzers equipped with up to five lasers and independent pinholes provide a large analytical capacity, although the complexity of the compensation matrices became increasingly challenging. FCM based on mass spectrometry allows using large panels of antibody combinations without the inconvenient of laborious compensations, however the technology is not widely available and requires significant investments. We therefore probed the capacity of spectral FCM to provide analytical ability similar to that of the most advance conventional FCM without requiring complex compensation strategies and using widely available fluorescent antibodies.

The results obtained with the two technologies (conventional versus spectral cytometry) can only be compared if the sensitivity of the two technologies is similar. We determined the staining index (S.I.) in One Comp beads stained with antibodies labeled with fluorochromes excited by the 405nm, 488nm, and 605nm lasers in two different conventional analyzers and in the spectral cytometer. Overall the S.I. (calculated by the formula: mean of fluorescence intensity (MFI) Pos population–MFI Neg population / 2 x SD Neg population) did not significantly differ between the three analyzers (S1 Fig). We found significant differences in the S.I. for Cy7-APC that was higher in the SP6800 than in instrument B while Pacific Blue S.I. was very high in all instruments although significantly higher in instrument B. We concluded that the S.I. obtained in spectral FCM is overall equivalent to that in conventional FCM and we proceeded to construct a panel of fluorescent-labeled antibodies to analyze immune cells in the mouse spleen. We chose fluorochromes exclusively excited by the 488 or by the 405 lasers, allowing using all emission wavelengths available in the 405 spectra and avoiding the mask inserted to blind the reflection of the 638nm laser.

**Fig 4:**
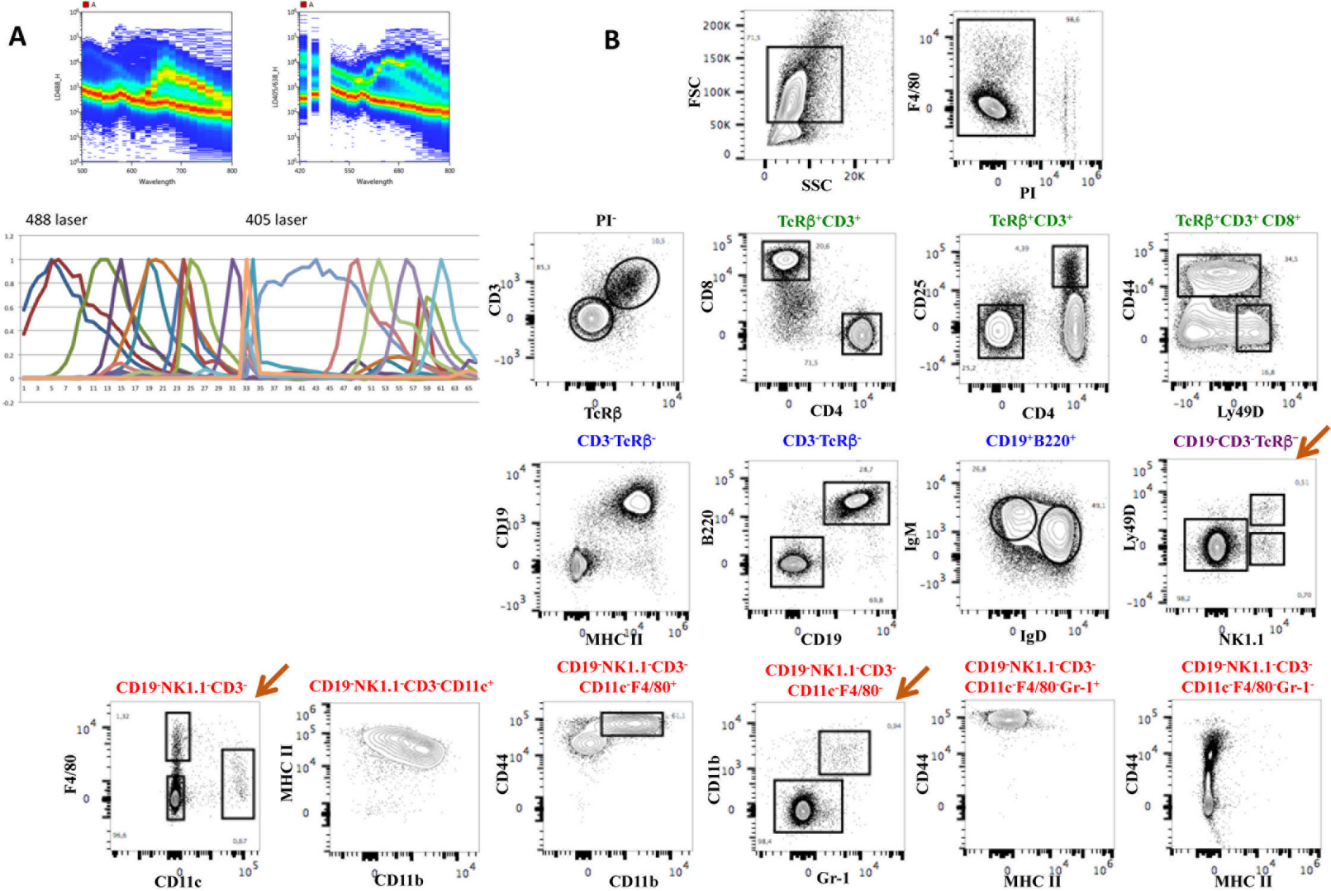
An 18-color antibody panel for the analysis of murine spleen cells. Multi-color antibodies panel with 18 different antibody-labeled fluorochromes, excited by the 488nm laser (blue) and by the 405nm laser (purple) (see Fig 5). The complex emission spectra of all fluorochromes are shown in the 488nm (upper left panel) and in the 405nm lasers (upper right panel) and in reference fluorochrome spectra shape with each colored curve corresponding to a different fluorochrome (lower panel)(**B**). **C**. The contour plots show the discriminative capacity of this multi-parametric analysis to separate the different populations of B, T, NK, dendritic or myeloid cells. The brown arrow shows rare subsets of NK and myeloid cells. The analysis was done in the FlowJo software after deconvolution.

Fig 4 shows the results obtained with the first antibody panel applied to splenic cells comprising 18 different antibodies recognizing different subsets of T, B, NK, dendritic and myeloid cells. The panel also included a viability dye (PI) as well as the size (FSC) and granularity (SSC) parameters (Fig 4). Fig 4A shows the complex emission spectra of all dyes combined in both lasers (upper panels) and the reference fluorochrome spectra shape (lower panel). After elimination of dead cells, the analysis (Fig 4B) shows that most CD3^+^ T cells express the TcRβ chain, have the expected CD4/CD8 distribution and proportion of CD25^+^ cells in CD4 T cells, while CD8 T cells show a normal distribution of CD44 and Ly49D expressing cells. CD19^+^ B cells co-express B220 and MHCII the majority of which also IgM and IgD, typical of mature splenic B cells. A subset of NK1.1^+^ NK cells that represent a small subset of the splenic lymphocytes co-expressed Ly49D. The remaining cells comprised a small subset of CD11c^+^ dendritic cells with high levels of MHC II some of which were also CD11b^+^. F4/80^+^ cells characteristic of tissue resident macrophages co-express CD11b and the highest levels of CD44 found in spleen. The remaining cells (CD19^-^Nk1.1^-^CD3^-^F4/80^-^CD11c^-^) could be subdivided by the expression of CD11b and Gr1, where the small subset of double positive cells also express high levels of CD44, likely corresponding to progenitors known to be present at low frequencies in the spleen, while a large fraction was negative for the three markers.

**Fig 5:**
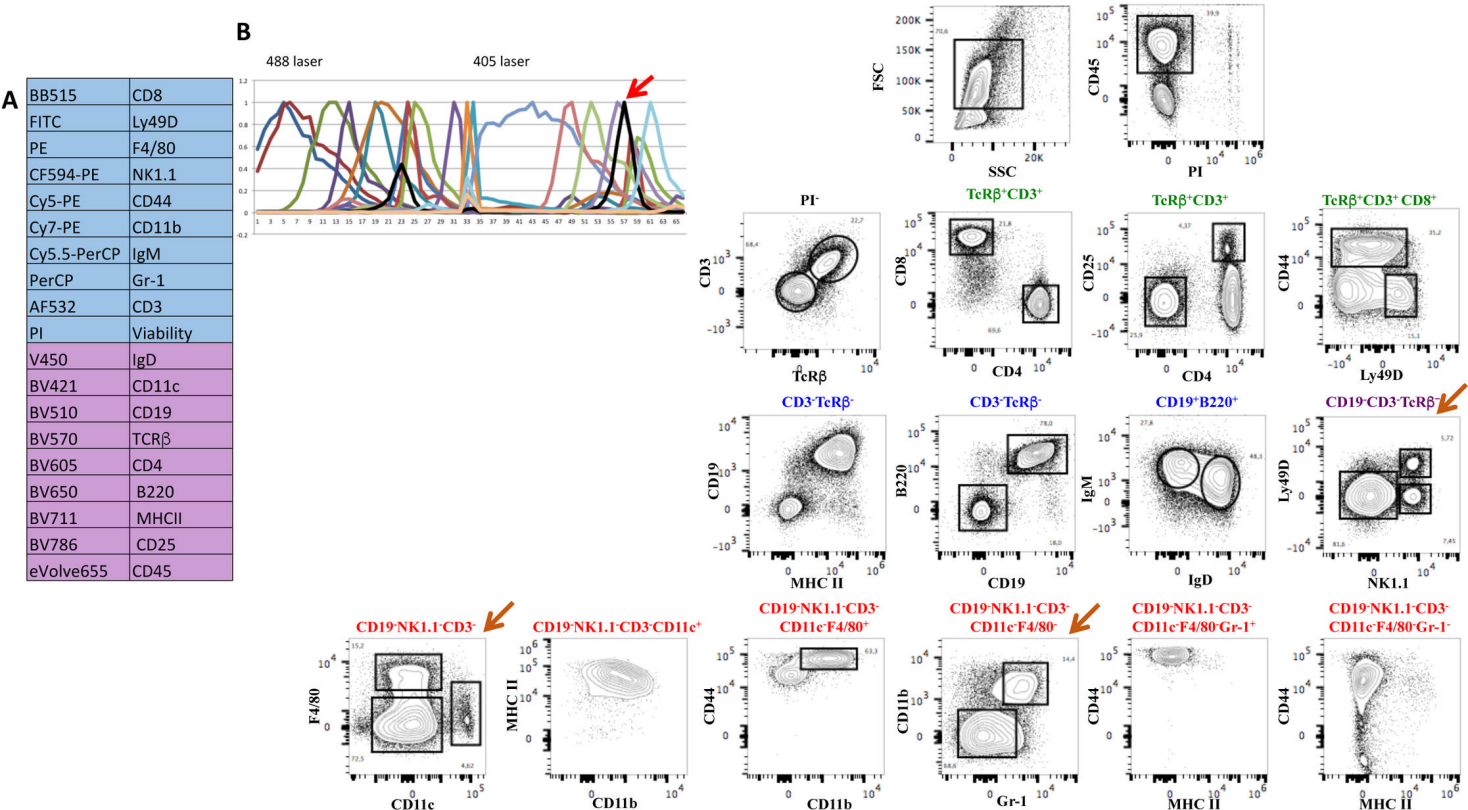
One additional fluorescent dye in the panel did not alter the discrimination of the splenocyte subsets. Mouse splenocytes were stained with the previous antibody combination to which CD45-eVolve655 was added. Panel **A** shows the list of antibodies and fluorochromes used in the experiment. **B**. The reference spectra of all fluorochromes are shown. The black line shows the reference spectrum of the newly added CD45-eVolve655 (pointed with a red arrow). **C**. The distribution of the populations was not altered by the introduction of an additional fluorochrome and rare cell subsets were now easily detected (brown arrow). The analysis was done in the FlowJo software after deconvolution.

We then tested whether introducing additional fluorochrome labeled antibodies would alter the discrimination of the fluorescent-labeled cell subsets. We chose to add CD45 because while the antibody panel could discriminate most splenic lymphocyte subsets, rare myeloid populations were difficult to analyze, even after acquisition of 1.5x10^6^ events, due to a large fraction of negative cells that likely correspond to non-hematopoietic cells (CD45^-^Ter119^-^) or erythrocytes (CD45^-^Ter119^+^). We used CD45-eVolve655 that we previously tested in combination with BV655. A second antibody panel that now assembles 19 fluorescent antibodies was tested and is shown in Fig 5A. Fig 5B shows the reference fluorochrome spectra shape of all spectra with the added fluorochrome in black (red arrow). Note that after gating for CD45^+^ cells, NK1.1^+^Ly49D^+^, as well as F4/80^+^ and CD11b^+^Gr1^+^ subsets appear as distinct well-discriminated populations (brown arrow). No differences in the shape of the populations or in the relative proportions of the cell subsets were detected after the introduction of an additional fluorochrome in this analysis. We thus showed the feasibility of establishing multi-parametric analysis in spectral FCM, using commercially available fluorescent labeled antibodies, with more parameters than those available in the most advanced conventional FCM, using only two excitation lasers and circumventing laborious compensation matrices.

### Auto-fluorescence does not interfere with the analysis in spectral FCM

FCM was historically used to analyze lymphocytes in the secondary lymphoid organs or hematopoietic progenitors in the bone marrow [8]. Adult hematopoietic or circulating immune cells, are easily dissociated into single cell suspensions, do not exhibit high adhesive properties and can be easily characterized due to the availability of a large collection of antibody specificities. In contrast, cells from many other tissues such as heart, intestinal epithelium, central nervous system or tumor infiltrates [9] are difficult to dissociate into single cells, a process that often requires enzymatic treatment [10]. Frequently cells isolated from solid organs display high auto-fluorescence and the choice of commercially available fluorescent antibodies discriminating these less well-defined cell types is reduced. We tested whether spectral FCM could improve the discrimination of auto-fluorescent populations, when they impair conventional FCM analysis in two distinct situations. First, when auto-fluorescent cells contaminate a preparation of lymphocytes and second, when the cells of interest for the analysis are auto-fluorescent.

**Fig 6:**
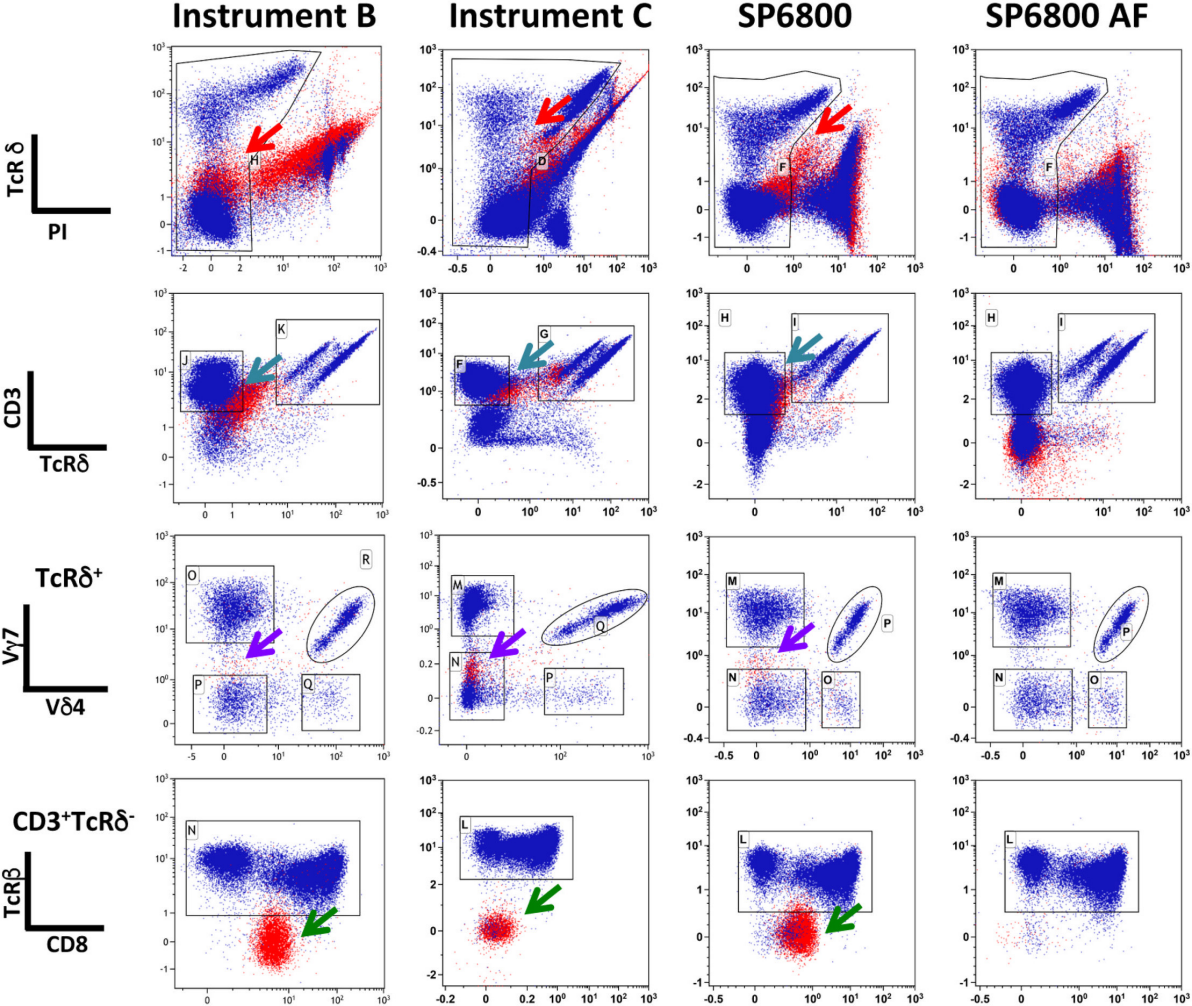
The presence of auto-fluorescent cells does not impair the detection of intra-epithelial lymphocytes, in spectral FCM. Small intestinal cells comprising epithelial cells and lymphocytes were stained with antibodies recognizing TcRδ-ΠE, TcRβ-Cy7-APC, X∆3-Pacific Blue, Vγ7-APC, CD8-FITC and Vδ4-Cy7-PE. PI was added in the FACS buffer before analysis. The acquisition of the data was done sequentially in the three instruments after appropriate quality control. Doublets were eliminated in the FSC-H/FSC-W. The analysis was done in the Kaluza 1.5 software. Plots show the different steps of the gating strategy. Cells within the FSC-A/SSC-A lymphocyte gate are labeled in blue while cells within the intestinal epithelial cell gate are labeled in red. Arrows point to different population distortions and auto-fluorescence. The first column from the left corresponds to data obtained in instrument B-LSR Fortessa (BD Biosciences); the second, to data obtained in instrument C-Cytoflex (Beckman Coulter); the third and fourth to data obtained in spectral FCM-SP6800 (Sony Inc.) without and with (SP6800 AF) auto-fluorescent management, respectively, after deconvolution.

Several T cell populations are found in the intestine within the epithelial cell layer [7,11]. Conventional isolation of these subsets includes mechanical disruption of the epithelium followed by a density gradient that separates lymphocytes from the majority of epithelial cells. However, this preparation is delicate, time consuming and results in cell loss. In order to improve quantification and to facilitate the experimental procedure we sought to test the potential of spectral FCM to discriminate populations of intestinal lymphocytes still mixed with a large fraction of epithelial cells. After mechanical disruption of the epithelial tissue, we directly stained the cell suspensions with antibodies that label subsets of αβ and γδ T cells usually found in the intestinal epithelium and analyzed them in two conventional cytometers and in spectral FCM. Fig 6 shows the results after following a similar gating strategy with elimination of dead cells gating on PI negative cells. The presence of auto-fluorescent cells is obvious in all conventional cytometers and in spectral cytometry, when the auto-fluorescence manager is inactivated (top panels-red arrows), resulting in a poor discrimination of the living cells. For clarity, in all plots cells within the FSC-A/SSC-A lymphocyte gate are shown in blue and cells with larger scatters (epithelial cell gate) are shown in red. Both conventional instruments were unable to resolve the CD3^+^ T cell subset (in Pacific Blue) (turquoise arrow) from the remaining cells. As a consequence, TcRβ^-^ cells (green arrow) contaminated the CD3^+^TcRγδ^-^ population, a subset never detected after separation of lymphocytes from epithelial cells and biologically improbable. In contrast, in spectral FCM after auto-fluorescence management the analysis of the different T cells subsets was not impaired by the presence of epithelial cells, the populations were well-separated and there were no TcR negative cells in the CD3^+^ gate. In order to better illustrate the way the deconvolution algorithm deals with auto-fluorescence we gated on non-lymphoid cells (S2 Fig) and followed them on similar gating strategy before and after activation of the auto-fluorescence manager, in the SP6800. The red arrow in the top panel shows auto-fluorescent cells that appear as TcRδ^+/-^ and as CD3^+/-^ in the PE and Pacific Blue channels (upper middle panel, blue arrow), respectively. They contaminate the APC (Vγ7) (lower middle panel purple arrow) but not the Cy7-APC (TcRβ) (lower panel green arrow) channels. The auto-fluorescence manager prevented the inclusion of these cells in the CD3^+^ TcRδ^+^ gate and thus excluded them from further analysis.

**Fig 7:**
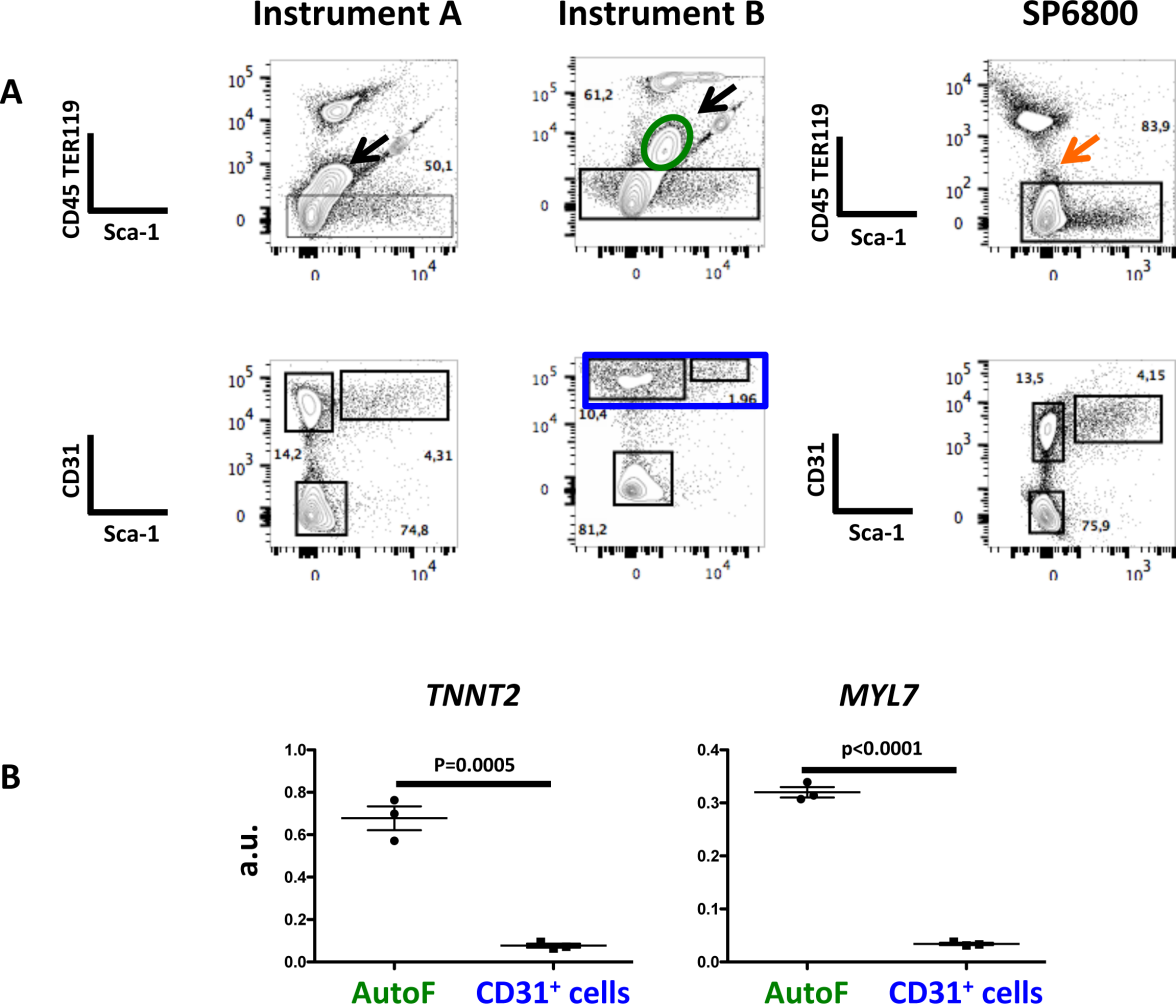
A large subset of auto-fluorescent cells in E17.5 cardiac cell preparations can be included in the fluorescence analysis, in spectral FCM. Embryonic hearts were isolated and sequentially digested with collagenase with mechanical dissociation at the end of each of seven consecutive digestion cycles. Cell suspensions were stained with antibodies anti TER119 and CD45-PE, Sca-1-Cy5-PE and CD31-APC. Acquisition of the data in the three instruments was done sequentially. Black arrows show auto-fluorescent cells in the two conventional cytometers while these cells are not detected as auto-fluorescent in spectral cytometry and can thus be analyzed for specific fluorescent staining. Instrument A is a Canto II and B a LSR Fortessa (BD Biosciences). B. Auto-fluorescent cells (within the elliptical gate in green) and CD31^+^ cells (rectangular gate in blue), taken as negative control, were sorted into lysis buffer and subjected to Q-RT-PCR that quantified cardiac troponin (*TNNT2*) [13] and atrial light chain-2 (*MYL7*) [14] transcripts specific for cardiomyocytes and not found in endothelial, stromal or hematopoietic cells. a.u. arbitrary units calculated relative to the expression of the house-keeping transcript GAPDH.

We then analyzed a sample of embryonic heart tissue that requires both mechanical disruption and enzymatic digestion to obtain single cell suspensions. We analyzed the expression of CD31 (an endothelial cell marker) and Sca-1 expressed by a fraction of endothelial and of stromal cells, after eliminating hematopoietic circulating cells (CD45^-^Ter119^-^) [12]. Fig 7 shows the comparative analysis in two conventional and in the spectral flow cytometer. A major population of auto-fluorescent cells (black arrow) appeared in conventional FCM and was excluded because they fluoresced in the CD45 and TER119 dump channel (PE) marking hematopoietic cells. In contrast, this auto-fluorescent population was not visualized as such in spectral FCM and was included in the CD45^-^TER119^-^ subset for further analysis. To show these were cardiac and not endothelial or hematopoietic cells expressing low levels of CD45, TER119 or CD31, we quantified the expression of transcripts specific of cardiomyocytes, on sorted cells. We show (Fig 7B) that they express high levels of cardiac muscle troponin T (*TNNT2*) [13] and atrial light chain-2 **(***MYL7*) transcripts [14], not found in CD31^+^ endothelial cells. Thus, a major subset of cardiac cells that escaped analysis in conventional FCM is included in the analysis in spectral FCM. We conclude that spectral FCM can attenuate the effects of auto-fluorescence either in cells analyzed for specific fluorescence staining or in by-stander populations that can contaminate the analysis. Therefore the spectral technology is a method of choice to characterize enzymatically treated cells isolated from solid organs.

## Discussion

Conventional FCM is based on the detection of photons emitted after excitation of fluorescent probes in precise ranges of wavelengths and as a consequence spilling over of emission spectra across detectors has to be mathematically subtracted, a procedure designated as compensation. As the number of detectors and of available fluorescent probes increased, compensation matrices became an increasingly complex, error prone and time-consuming procedure. Moreover, because fluorescence is subtracted there is loss of photons and the choice of a panel of fluorescent antibodies needs careful attention to avoid combining antibodies with close values of PEm. Depending on the set of filters and mirrors in a given configuration, the combination of fluorochromes can require excessive compensations that distort the visual representation of the populations and decreases the fluorescence intensity. Thus, the discrimination of the populations can be impaired and generate non-existing cell subsets. An additional source of artifacts is auto-fluorescence that also impairs analytical capability because it impairs determining the specificity of the fluorescence signal.

Spectral FCM and data processing by the unmixing deconvolution algorithm deals with some of these problems. First, because the whole spectrum is analyzed, rather than a short range of emission, we could combine fluorochromes with close PEm, provided different spectral shape. Second, because the algorithm used for analysis treats auto-fluorescence as an independent parameter, allows specific fluorescence detection in auto-fluorescent cells. The SP6800 compares spectra rather than PEm and automatically deals with deconvolution for overlapping emissions.

Here we compared the properties of spectral FCM in allowing combining fluorescent probes that cannot usually be discriminated and in resolving auto-fluorescence, with those of conventional FCM. Although the deconvolution algorithm does not use mathematical subtraction to resolve similar emission peaks we found the S.I. did not significantly differ between the two types of cytometry analysis thus allowing direct comparison of the results. Importantly, not only did spectral FCM separate fluorochromes with close PEm, but also it discriminated fluorochromes with virtually identical PEm provided that they differ in the shape of the emission spectra. Importantly, spectral FCM could discriminate FITC, YFP and GFP labeled cells although the three fluorochromes analyzed together led to somewhat lower resolution of the plots. We assembled a panel of 19-fluorochromes to analyze immune cells with only two lasers (488nm and 405nm) using conventional fluorescent antibodies with discrimination of subsets comparable to that of conventional FCM. Of note we show that the sequential addition of new fluorochromes do not affect the resolution of the populations.

The definition of surface phenotypes of hematopoietic progenitors allowed the identification of the first adult stem cell compartment that became the paradigm in stem cell biology [8]. As a consequence, developmental and stem cell biologists have been attempting to replicate this model in other adult and embryonic tissues not only in mammals but also in other animal models. However, these efforts have been hampered by the difficulty in analyzing cell suspensions issued from solid organs that require mechanical disruption and/or enzymatic digestion because either auto-fluorescent cells mask the properties of the populations of interest or the analyzed cells exhibit themselves high levels of auto-fluorescence.

We tested two different situations where auto-fluorescence can impair the analysis, one, where lymphocytes are contaminated with epithelial cells and the other, where auto-fluorescent cardiac cells are analyzed. We found that unlike in conventional FCM, spectral cytometry allowed discriminating all lymphocyte subsets even when they represent minor subsets. This was due to the deconvolution algorithm that subtracted the auto-fluorescence that, depending on the cell type, appeared in different channels.

The regenerative capacity of adult cardiomyocytes has been a matter of a long debate [15–17]. This is in part due to the absence of a strategy to discriminate and isolate distinct subsets of viable cells in the heart, thus impairing efforts in recognizing lineage relationship and characterizing tissue compartments with potential regenerative capacity. Only a multi-parametric analysis will define a unique combination of markers that discriminates the different cardiac subsets along development and FCM provides the capacity to detect rare subsets because of large numbers of cells that can be analyzed. Our analysis of late embryonic hearts (E17.5) isolated at a period when all cardiac subsets (cardiomyocytes, stromal and endothelial cells) are found indicates that a large fraction of auto-fluorescent cells that would escape examination in conventional FCM appears integrated in the non-hematopoietic viable cardiac cell compartment in spectral FCM. Recent advances in tumor biology using constitutively expressed fluorescent dyes that discriminate stromal and tumor cells [18] allowed the functional characterization of stroma-tumor cell interactions and their role in tumor progression and metastatic capacity [9,19]. The capacity to analyze in spectral FCM dyes with similar peak of emission while integrating auto-fluorescence of stromal and tumor cells could substantial widen this analysis. In recent years FCM based on mass spectrometric provided an analytical power higher than that obtained with conventional FCM and without the need for compensation matrices. We provide evidence that there is an alternative approach combining high analytical capacity, absence of complex compensation matrices, no impaired discrimination of cell subsets due to auto-fluorescence and no additional financial commitments still required for mass spectrometry FCM. Spectral FCM appears to be a method of choice for the analysis of cells obtained from a wide variety of solid tissues usually not amenable to conventional FCM to be used in tumor, developmental and stem cell biology.

## Supporting Information

S1 Fig The staining index (S.I.) is similar in conventional and spectral cytometry. One Comp beads (eBiosciences) were stained with antibodies marked with FITC, Cy7-PE (excitation 48nm), APC, Cy7-APC (excitation 650nm), Pacific Blue and V500 (excitation 405nm). They were analyzed in the two conventional and in the spectral FCM. The S.I. for PE and Cy5-PE were also determined and were not different in the three analyzers (not shown). The S.I. was calculated by the formula MFI of the positive population-MFI of the negative population/2 x standard deviation of the negative population. Note that only instrument B is equipped with a dedicated Yellow/Green laser for the detection of PE, Cy5-PE and Cy7-PE while in instrument A and in the SP6800 detection is done by the 488 laser.
(TIF)

S2 Fig The auto-fluorescence management of the deconvolution algorithm in spectral FCM. Small intestinal cells comprising epithelial cells and lymphocytes were stained with antibodies recognizing the TcRδ-PE, TcRβ-Cy7-APC, CD3-Pacific Blue, Vγ7-APC, CD8-FITC and Vδ4-Cy7-PE as in Fig 6. PI was added in the FACS buffer before analysis. Data acquired in the SP6800 was analyzed in the Kaluza 1.5 software after deconvolution. The left plots (SP6800) show the data excluding lymphocytes and enriched for epithelial cells, gated in FSC: SSC, analyzed before and after (right plots-SP6800 AF) activation of the auto-fluorescence manager. Arrows show auto-fluorescent cells in the corresponding channels.
(TIF)

S1 Table List of the antibodies used in this study. (EPS)
