# Supplementary figures and images for: Spectral Cytometry Has Unique Properties Allowing Multicolor Analysis of Cell Suspensions Isolated from Solid Tissues
Source: PLoS One. 2016 Aug 8;11(8):e0159961. doi: 10.1371/journal.pone.0159961 (PMC4976887; doi:10.1371/journal.pone.0159961)

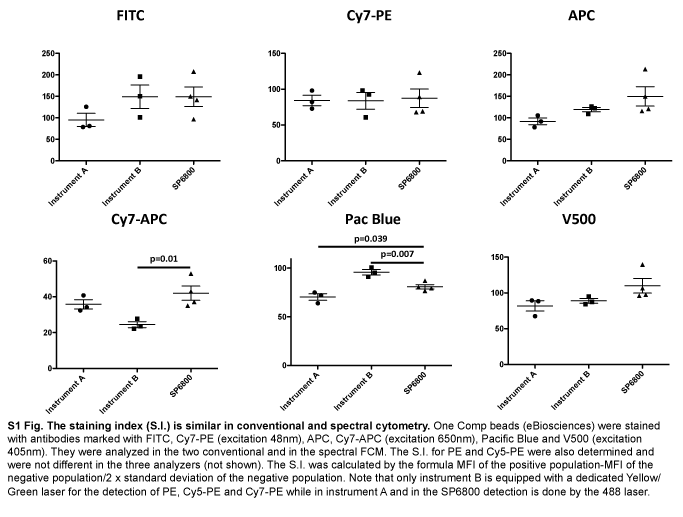

Supplement: S1 Fig — One Comp beads (eBiosciences) were stained with antibodies marked with FITC, Cy7-PE (excitation 48nm), APC, Cy7-APC (excitation 650nm), Pacific Blue and V500 (excitation 405nm). They were analyzed in the two conventional and in the spectral FCM. The S.I. for PE and Cy5-PE were also determined and were not different in the three analyzers (not shown). The S.I. was calculated by the formula MFI of the positive population-MFI of the negative population/2 x standard deviation of the negative population. Note that only instrument B is equipped with a dedicated Yellow/Green laser for the detection of PE, Cy5-PE and Cy7-PE while in instrument A and in the SP6800 detection is done by the 488 laser. (TIF) [file pone.0159961.s001.tif]

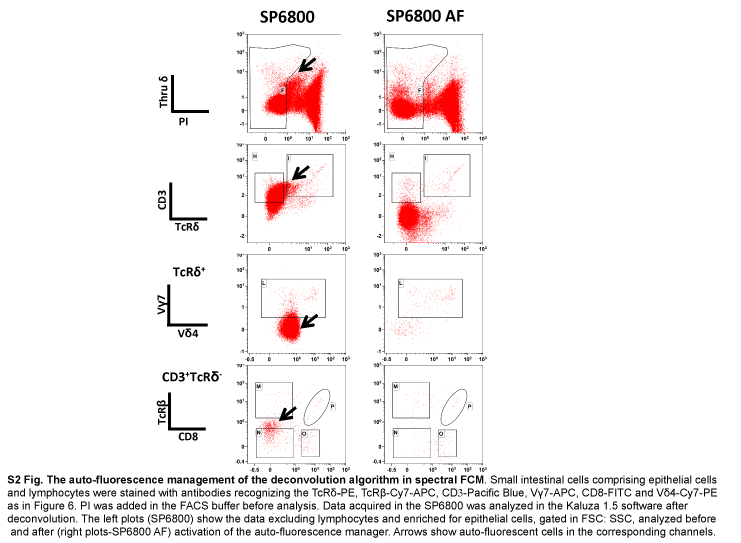

Supplement: S2 Fig — Small intestinal cells comprising epithelial cells and lymphocytes were stained with antibodies recognizing the TcRδ-PE, TcRβ-Cy7-APC, CD3-Pacific Blue, Vγ7-APC, CD8-FITC and Vδ4-Cy7-PE as in Fig 6. PI was added in the FACS buffer before analysis. Data acquired in the SP6800 was analyzed in the Kaluza 1.5 software after deconvolution. The left plots (SP6800) show the data excluding lymphocytes and enriched for epithelial cells, gated in FSC: SSC, analyzed before and after (right plots-SP6800 AF) activation of the auto-fluorescence manager. Arrows show auto-fluorescent cells in the corresponding channels. (TIF) [file pone.0159961.s002.tif]
